# RAB26 promotes prostate cancer progression via the MAPK/ERK-TWIST1 signaling axis

**DOI:** 10.1016/j.gendis.2025.101689

**Published:** 2025-05-22

**Authors:** Hexi Wang, Simin Liang, Xiaoyi Du, Guozhi Zhao, Yuanyuan Bai, Junwu Li, Haoyu Xu, Senlin Peng, Ye Yuan, Wei Tang

**Affiliations:** a Department of Urology, The First Affiliated Hospital of Chongqing Medical University, Chongqing 400010, China; b Department of Urology, Suining First People's Hospital, Suining, Sichuan 629000, China; c Department of Urology, Chongqing Thirteenth People's Hospital, Chongqing 400053, China

**Keywords:** Cancer stem cells, EMT, Prostate cancer, RAB26, TWIST1

## Abstract

RAB26 is important in the regulation of membrane trafficking and cell motility. Recently, RAB26 has received increasing attention in cancer research. However, the functional role of RAB26 in prostate cancer (PCa) remains to be elucidated. Single-cell RNA sequencing data (GSE141445) analysis indicated that RAB26 was widely expressed in PCa cells, especially in luminal cells and basal cells. High RAB26 expression in patients was found to be significantly associated with advanced pathological stage, Gleason score, and poor prognosis. Furthermore, our experimental results showed that RAB26 promoted the proliferation, migration, and invasion of PCa cells, as well as influenced the stemness of PCa cells *in vitro*. Besides, the transcription sequence indicated that RAB26 might promote the metastatic potential of PCa by promoting epithelial–mesenchymal transition through cascades of MAPK/ERK pathways. Finally, we found that RAB26 activated TWIST1 expression and consequently induced epithelial–mesenchymal transition, based on its interaction with the TWIST1 promoter. In conclusion, RAB26 promotes the aggressive progression of PCa and stemness of tumor cells, which is an independent biomarker for the prognosis of PCa.

## Introduction

The second most common type of cancer in men is prostate cancer (PCa), which affects one in nine men in the United States during their lifetime.^1^ Although early detection of PCa has improved recently, treatment of late-stage disease remains ineffective and limited.^2^ Radical prostatectomy can indeed cure high-risk PCa, but a subset of patients may experience prostate-specific antigen (PSA) recurrence and suffer from PCa death as well.^3^ Androgen deprivation therapy is the primary treatment for PCa with an advanced stage, but nearly all patients progress to castration-resistant PCa.^4^^,^^5^ Further, there is little curative effect from the therapeutic approaches for patients with advanced and metastatic disease.^6^ Therefore, understanding what drives prostate carcinogenesis and how cancer cells grow and metastasize may help develop new therapeutics that may succeed when standard treatments fail.

Cancer stem cells are capable of self-renewal and differentiation and can trigger tumor growth, metastasis, and recurrence after chemotherapy.^7^ In many previous studies, prostate cancer stem cells (PCSCs) were demonstrated to be present in malignant prostate tumors.^8^^,^^9^ PCSCs do not express the androgen receptor, and their growth does not depend on androgen receptor.^10^ A critical role for PCSCs is not only in tumor initiation but also in advanced malignant progression to castration resistance and metastasis, which may be related to their insensitivity to androgen deprivation therapy and phenotypic plasticity.^11^ Present studies have demonstrated that PCSCs confer castration and recurrence by activating multiple signaling pathways, such as those regulated by WNT/β-catenin, phosphoinositide 3-kinase (PI3K)/protein kinase B (AKT), and nuclear factor kappa-B (NF-κB) pathway.^12^ Therefore, cancer stem cells are becoming a prime candidate for studying the molecular mechanisms that control tumorigenesis and metastasis.^13^

RAB proteins are small GTPases that belong to the Ras superfamily. It is known that RAB proteins are critical regulators of vesicular trafficking. They provide vesicle motility, tethering, and fusion mechanisms within secretory and endocytotic pathways.^14^ During cycling, RAB proteins cycle between GDP-bound states, GTP-bound states, and membrane-bound states.^15^

In recent years, evidence has accumulated that changes in RAB protein expression are linked to cancer progression.^16^ For example, RAB3D, RAB7, RAB11, RAB23, and RAB25 promote cancer cell proliferation, migration, invasion, and metastasis by disrupting the homeostasis of intracellular signal transduction and vesicular trafficking in PCa.^17^, ^18^, ^19^, ^20^, ^21^, ^22^ However, only a few fractions of RAB proteins are implicated in cancer stemness. There is only a few evidence concerning a direct link between RAB GTPases and cancer stem cell status. For example, RAB37 mediates exocytosis of secreted frizzled-related protein 1 (SFRP1) to inhibit WNT signaling and thus suppress lung cancer stemness.^23^ Furthermore, by activating the extracellular signal-regulated kinase 1/2 (ERK1/2) signaling pathway, RAB2A was found to promote breast cancer stem cells and tumorigenesis *in vitro* and *in vivo*.^24^

RAB26 is a member of the RAB family group Ⅲ, and others include RAB27A, RAB27B, RAB37, and RAB3, which are commonly found in exocrine cells.^25^ Among these, RAB26 and RAB3D are direct transcriptional targets of muscle, intestine, and stomach expression 1 (MIST1) that regulate exocrine granule maturation.^26^ It has been shown in several studies that RAB26 is not only involved in regulating membrane trafficking but also affects cell function. Small nuclear ribonucleoprotein polypeptides B and B' (SNRPB) may regulate the tumorigenic potential of non-small cell lung cancer by modulating RAB26 expression, not by relying on RAB26's autophagic function.^27^ At the same time, by interacting with autophagy-related 16-like 1 (ATG16L1), RAB26 directs synaptic vesicles to undergo autophagy and inhibits migration and invasion of invasive breast cancer cells.^28^ A recent study has shown that RAB26 regulates the degradation of phosphorylated Src to enhance adhesion junction integrity.^29^ Therefore, the role of RAB26 in cancer deserves further study. Despite this, RAB26 has not been studied in PCa, which requires further investigation.

The present study aimed to elucidate the significance of increased RAB26 expression and the role of enhanced RAB26 production in the growth regulation of PCa. Also, the study sought to characterize the factors responsible for its up-regulation and the downstream effectors involved. As a result, we found that RAB26 was highly expressed in PCa. Down-regulation of RAB26 expression *in vitro* can suppress cell proliferation, invasion, and migration, and promote apoptosis. Studies have revealed that up-regulated RAB26 expression is associated with a poor prognosis in patients with PCa. Moreover, our findings indicated that RAB26 was preferentially enriched in PCSCs and enhanced the stem cell-like traits of PCa cells. Our study indicated that the knockdown of RAB26 significantly suppressed the mitogen-activated protein kinase (MAPK)/ERK signaling pathway. Further investigation revealed that RAB26 activated the MAPK/ERK signaling pathway, with increased levels and nuclear translocation of p-ERK. Also, we found that RAB26 facilitated p-ERK-mediated nuclear translocation of Twist family BHLH transcription factor 1 (TWIST1), regulating epithelial–mesenchymal transition (EMT) and stemness in PCa cells. Additionally, TWIST1 could activate RAB26 gene transcription by binding to its promoter.

## Materials and methods

### Patient information and tissue specimens

The specimens included twelve pairs of PCa tissue and adjacent non-tumor tissue from individuals diagnosed with PCa at the First Affiliated Hospital of Chongqing Medical University. During the period from 2020 to 2022, 172 paraffin-embedded, archived, histopathologically and clinically diagnosed clinical PCa samples were obtained at the First Affiliated Hospital of Chongqing Medical University. None of the above patients received androgen deprivation therapy, radiotherapy, or chemotherapy before the operation. Before specimen collection, all patients were informed that the PCa tissue obtained through surgery would be used for scientific research, and they signed an informed consent form, which was kept on record. An ethics committee of the First Affiliated Hospital of Chongqing Medical University approved the study protocol following the Helsinki Declaration (No. K2023-279).

### Cell lines and cell culture

RWPE-1 and 22RV1 cells were kind gifts from the research group of Prof. XIAO-HOU Wu (the department of Urological Department of the First Affiliated Hospital of Chongqing Medical University). LNCaP-clone-FGC, PC-3, and DU145 cells were purchased from Procell Life Science & Technology Co., Ltd. RWPE-1 cells were cultured in a keratinocyte medium (Sciencell, USA). LNCaP-clone-FCG and 22RV1 cells were cultured in RPMI 1640 medium supplemented with 10% fetal bovine serum and 1% penicillin/streptomycin. DU145 cells were cultured in MEM medium supplemented with 10% fetal bovine serum and 1% penicillin/streptomycin. PC-3 cells were cultured in Ham's F12K medium supplemented with 10% fetal bovine serum and 1% penicillin/streptomycin.

### Western blotting

Using standard methods, western blotting was performed. Briefly, cells were collected, washed in phosphate-buffered saline solution (PBS), and lysed in lysis buffer. SDS-PAGE was used to resolve the proteins in lysates, and then 0.22 μm PVDF membranes were used to transfer them. To block PVDF membranes, 5% dry milk was applied for 1 h. Incubation of primary antibodies took place at 4 °C overnight. Antibodies included anti-RAB26 (14284-1-AP, 1:1000; Proteintech), anti-β-tubulin (EM0103, 1:5000; Huaian), anti-E-cadherin (20874-1-AP, 1:20000; Proteintech), anti-N-cadherin (22018-1-AP, 1:2000; Proteintech), anti-vimentin (ET1610-39, 1:20000; Huaian), anti-p-Erk1/2 (#4370, 1:2000; CST), anti-ERK (ab184699, 1:10000; Abcam), anti-p-MAPK (#4511, 1:1000; CST), anti-MAPK (#4370, 1:1000; Biomake), anti-β-actin (66009-1-lg, 1:20000; Proteintech), anti-lamin A/C (ET7110-12, 1:1000; Huaian), anti-TWIST1 (507314, 1:1000; Zenbio), anti-snail1 (3879, 1:1000; CST), anti-slug (4719, 1:1000; CST), anti-GM130 (R380777, 1:100; Zenbio), anti-MMP2 (ET7110-12, 1:1000; Huaian), and anti-MMP9 (ET7110-12, 1:1000; Huaian).

### RNA extraction, reverse transcription, and real-time PCR

The total RNA from cultured cells was extracted using an RNA-Quick purification kit (ES Science, Shanghai, China). To obtain cDNA, total RNA was reverse-transcribed using the PrimeScript™ RT Reagent Kit with Eraser for gDNA (Takara, China). Then, real-time PCR was carried out using SYBR Premix Ex Taq (Takara, China), and mRNA expression levels were normalized to the levels of β-actin. To calculate the expression of each gene relative to another, the comparative method (CT) had to be used (ΔΔCT method). All primer sequences are listed in Table 1.

**Table 1:** Real-time PCR primers.

Name	Sequence (5′ to 3′)
RAB26-Up	CATCTCCACCGTAGGCATTGACTTC
RAB26-Down	CAGCAGCAGAGCATGAGCATCC
NANOG-Up	TCCAACATCCTGAACCTCAGCTA
NANOG-Down	AGTCGGGTTCACCAGGCATC
OCT4-Up	GTCCGAGTGTGGTTCTGTA
OCT4-Down	CTCAGTTTGAATGCATGGGA
SOX2-Up	GTGAGCGCCCTGCAGTACAA
SOX2-Down	GCGAGTAGGACATGCTGTAGGTG
Bmi1-Up	TCGTTGTTCGATGCATTTCT
Bmi1-Down	CTTTCATTGTCTTTTCCGCC
c-Myc-Up	CCTCCACTCGGAAGGACTATC
c-Myc-Down	TGTTCGCCTCTTGACATTCTC
GAPDH-Up	GCACCGTCAAGGCTGAGAAC
GAPDH-Down	TGGTGAAGACGCCAGTGGA
ACTIN-Up	CCTTCCTGGGCATGGAGTC TGATCTTCATTGTGCTGGGTG
ACTIN-Down	TGATCTTCATTGTGCTGGGTG
CDH1-Up	CGAGAGCTACACGTTCACGG
CDH1-Down	GGGTGTCGAGGGAAAAATAGG
N-cadherin-Up	TCAGGCGTCTGTAGAGGCTT
N-cadherin- Down	ATGCACATCCTTCAGTAAGACTG
VIM-Up	GACGCCATCAACACCGAGTT
VIM-Down	CTTTATCGTTGGTTAGCTGGT
Twist1-Up	GTACATCGACTTCCTCTACCAG
Twist1-Down	CATCCTCCAGACCGAGAAG
Snail-Up	TCGGAAGCCTAACTACAGCGA
Snail-Down	AGATGAGCATTGGCAGCGAG
Slug-Up	CGAACTGGACACACATACAGTG
Slug-Down	CTGAGGATCTCTGGTTGTGGT

### CCK-8 assay

Cell viability was measured using the CCK-8 Assay Kit (Biosharp, China) according to the manufacturer's instructions. A density of 5 × 10^3^ cells per well was applied to 96-well plates containing 200 μL of medium. Ten microliters of CCK-8 reagent were added to 96-well plates and tested with absorbance at 450 nm, every 24 h.

### EdU assay

Cell proliferation was assessed using an EdU kit (BeyoClick™ EdU Cell Proliferation Kit with Alexa Fluor 555, C0075S, Beyotime, China). Briefly, cells were seeded into 96-well plates at 3000 cells per well. After EdU treatment, the EdU staining was performed following the kit instructions. Image capture was done using a light microscope under five randomly selected fields of view. Using the number of EdU-positive cells multiplied by the total number of cells in each field of view, we calculated the percentage of proliferating cells.

### Sphere formation assay

To determine the capacity for sphere formation, an assay for sphere formation was conducted. Culture media consisted of 1640/Ham's F-12K, containing 20 ng/mL epidermal growth factor (EGF; PeproTech, USA), 20 ng/mL fibroblast growth factor, and B27 (50 × ) (Gibco, Gaithersburg, MD, USA). The cells were plated sparsely at a density of 2000 cells/well in Corning ultralow attachment plates (6 wells). Incubation conditions were 37 °C, 5% CO_2_, and 95% humidity for 7–14 days. After culture for seven days, inverted microscope-camera images of spheres were captured.

### Wound healing assay

Each of the three cell lines was tested with an identical scratch area to determine migration. Briefly, cells (1 × 10^6^) were seeded in 6-well plates, in duplicate. 200-μL pipette tips were used to make linear scratch wounds on confluent monolayers after adhesion. During the migration process, a serum-free medium was used to maintain the cells and allow them to be incubated for 24 h. Inverted microscopes were used to take photographs at 0, 12, and 24 h after the wound was closed. Using Photoshop software (Adobe, San Jose, CA, USA), we measured the distance between the scratch and the surface of the image.

### Transwell assay

*In vitro* migration and invasion assays have been used to evaluate the functional characteristics of cell migration and invasion *in vitro*. In the upper compartment of the transwell permeable chambers (JET BIOFUL, 8 μm, Guangzhou, China), cells were plated, the chamber was coated without Matrigel for migration, and it was coated with Matrigel (Corning, USA) for invasion detection.

Serum-free medium was added to the upper chambers, whereas a medium containing 20% fetal bovine serum was added to the lower chambers. Then, 4% paraformaldehyde was used to fix and 0.1% crystal violet to stain the lower chamber cells. Finally, we counted the number of invaded and migrated cells under the light microscope.

### Clone formation experiment

Using a plate clone formation assay, we measured the clone formation rate. Cells were seeded at a density of 1000 per well in 6-well plates at 37 °C and incubated with 5% CO_2_. After 14 days, the cells were fixed with 4% paraformaldehyde for 15 min and stained with crystal violet for 30 min. Finally, a microscope was used to examine it, and pictures were taken.

### Transfection

A lentiviral vector for RAB26 knockdown and overexpression (shRAB26 and OE-RAB26) was purchased from GenePharma (Shanghai, China). The corresponding sequences were as follows: OE-RAB26: NCBI Reference Sequence: NM_014353.5GenBank Graphics > NM_014353.5: 160-930 *Homo sapiens* RAB26, member RAS oncogene family (RAB26), transcript variant 1, https://www.ncbi.nlm.nih.gov/nuccore/NM_014353.5; shRNA-RAB26-1: 5′-CCGGCTGCATGATTACGTTAA-3'; shRNA-RAB26-2: 5′-GCATTGACTTCCGGAACAAAG-3'. To form stable cell lines, we transfected lentiviruses with multiplicities of infection from 10 to 100 on each cell line and selected them with puromycin 48 h after transfection.

### Plasmid transfection

TWIST1 overexpression plasmid (pcDNA3.1-c-TWIST1) was constructed by Wuhan Jinkairui Bioengineering Co., Ltd. (China). Polyplus jetPRIME reagent was used to transfect plasmids into cells. To transfect plasmids, cells were seeded to 70%–90% confluency. For transfection, we added 2 μg of vector and 4 μL of jetPRIME transfection reagent to 200 μL of jetPRIME buffer at room temperature and incubated them for 15 min. After that, cells were incubated at 37 °C in a CO_2_ incubator for 48 h before being tested for transgene expression.

### Nucleocytoplasmic separation assay

Separating cytoplasmic and nuclear proteins was accomplished using the Nuclear and Cytoplasmic Protein Extraction Kit according to the manufacturer's protocol. To summarize, cells were harvested using trypsinization, washed once with PBS, and resuspended in 1 mL of PBS. Afterward, the cell pellet was resuspended in ice-cold cytosolic buffer and incubated for 15 min on ice. In centrifugation, the supernatant contained cytoplasmic fractions, and the precipitate contained nuclear pellets. Using a cell disruption buffer, the nuclear pellets were suspended. After that, the experiment proceeded similarly to the western blotting.

### Dual-luciferase reporter assay

Luciferase reporter plasmids were constructed from TWIST1 dual-luciferase vectors (Jinkairui Bioengineering, Wuhan, China). For the dual-luciferase reporter assay, 293T cells were used. The sequences of RAB26 containing the wild-type (WT) or mutant (Mut) binding site of Twist1 were devised and synthesized by Jinkairui (Wuhan, China). To construct a luciferase reporter gene vector containing TWIST1 promoter, the full-length RAB26 promoter containing wild or mutant type was respectively cloned into pGL3-basic vectors (Jinkairui, Wuhan, China), and co-transfected with or without TWIST1 overexpression vector later. After 48 h of incubation, the activities of firefly and Renilla luciferase were measured using a dual-luciferase reporter assay kit (YEASEN, Shanghai, China).

### Transcriptome analysis (RNA sequencing)

As part of the transcriptome analysis, Lianchuan Biotechnology Co., Ltd. (China) carried out the entire procedure. With the assistance of TRIzol, RNA was extracted from the harvested cells. To obtain mRNA, we purified the total RNA and decomposed it into 200-300 bp fragments. Following fragmentation, random hexamer primers were used to synthesize first-strand cDNA, followed by the synthesis of second-strand cDNA. In the end, PCR-amplified cDNA was prepared. To generate raw data, RNA samples were sequenced by the Illumina NovaseqTM 6000 platform, and the sequencing mode was PE150.

### Flow cytometric analysis

Analysis of cell apoptosis and cell cycle progression was performed using flow cytometry. Briefly, following digestion, the cells were collected and washed once in PBS. After resuspension, the cells were subsequently prepared as single-cell suspensions. We washed the cells and analyzed them on a FACS system (BD, San Jose, CA, USA) after 30 min.

### Cell immunofluorescent staining

Three washes of PBS were administered to the cultured cells before they were stained with an immunofluorescent stain. Afterward, cells were fixed in pre-cooled 4% paraformaldehyde at 4 °C and rinsed three times with PBS. The cells were then permeabilized with 0.5% Triton X-100 for 20 min and blocked with an immunostaining closure solution for 90 min at room temperature. Afterward, we incubated the primary antibody at 4 °C overnight in a humidified chamber. Following three washings with PBS, the cells were incubated with the secondary antibody for 60 min, washed, and then incubated with DAPI (DA0004, Leagene Biotecnology, China) for 5 min in the dark. Lastly, photographs were captured under a fluorescence microscope (Olympus, Japan).

### Confocal immunofluorescence staining

The confocal immunofluorescence staining steps were the same as immunofluorescence staining, but the fluorescence labeling was performed using the FlexAble CoraLite® Plus 555 Antibody Labeling Kit (KFA002, Proteintech, China) for the RAB26 and TWIST1 and the FlexAble CoraLite® Plus 647 Antibody Labeling Kit for Rabbit IgG (KFA003, Proteintech, China) for the p-ERK and GM130 as per the manufacturer's protocol.

### Immunohistochemistry analysis and hematoxylin-eosin staining

According to standard protocols, immunohistochemistry analysis and hematoxylin-eosin staining were performed. PV9001 and DAB staining kits were purchased from Beijing Zhongshan Golden Bridge Biotechnology Co., Ltd., Solaibao (Beijing, China). Briefly, paraffin sections were dewaxed and rehydrated, and the antigen was extracted by heating in EDTA buffer (pH 9.0) for 15 min in a pressure cooker, followed by incubation at room temperature for 10 min with endogenous peroxidase blocker and then rinsing with PBS buffer three times in the next 3 min. Then, appropriate amounts of primary antibody were added and incubated at 4 °C overnight. Next, the sections were washed 3 times with PBS and then incubated at 37 °C for 20 min after the addition of a reaction enhancer dropwise, followed by incubation with an anti-rabbit IgG antibody at 37 °C for another 20 min. In addition, a new solution for developing DAB colors was added. The dyed fabric should be washed with tap water after dyeing, incubated for 20 s in the hematoxylin staining solution, and then differentiated, rinsed, and put back in the blue color. In the final step, slides were dehydrated with gradient ethanol, permeabilized with xylene, and then sealed and photographed.

### TUNEL staining

The TUNEL staining was carried out using a TUNEL kit from Servicebio, Wuhan, China (CF488), following the manufacturer's instructions. In brief, following dewaxing and rehydrating, immunostaining was performed. Afterward, these sections were incubated at 37 °C for 20 min with 200 μg/mL proteinase K working solution, and then PBS was applied 3 times. Following the addition of 100 mL of equilibration buffer, 50 μL of TdT reaction mix was added to the tissue section, and incubation continued at 37 °C in the dark for 1 h. Nuclei were stained with DAPI at room temperature for 8 min after being rinsed with PBS three times. To conclude the procedure, the sections were sealed with an anti-fluorescence quencher and observed and photographed with a fluorescence microscope (Olympus).

### Tumor growth in xenografts

All animal experiments were conducted at Chongqing Medical University and were approved by the Ethics Committee of Chongqing Medical University (No. IACUC-CQMU-2023-11043). Four-week-old male BALB/c(nu/nu) nude mice were purchased from Weitonglihua Biotechnology (Beijing). Institutional guidelines were followed in the care and treatment of experimental animals. The mice were randomly allocated to each group. 2 × 10^7^ LNCaP-vector cells and LNCaP-shRAB26 cells mixed with Matrigel (1:1) were subcutaneously injected into the back of each mouse near the right forelimb. Following injection, tumor volumes were measured every 7 days with calipers and calculated using the equation: volume = length × width^2^ × 0.5. Eight weeks after the mice were sacrificed, their tumor sizes were measured, and their weights were recorded. A metastatic xenograft model was developed using nude mice injected with LNCaP cells. Tumor burden was measured using a Xenogen IVIS Spectrum Imaging System with bioluminescence imaging.

### Bioinformatic analysis

We first used the “Seurat” package to preprocess the PCa single-cell sequencing data set from GSE141445. Preprocessing includes quality control to exclude aberrant cells due to sequencing depth, gene expression diversity, and cell cycle differences. Principal component analysis was used to select specifically highly expressed genes for each cell subpopulation, and then the cells were clustered using Seurat's “FindNeighbors” and “FindClusters” functions, with the clustering resolution set to 0.1. Seurat's “RunUMAP” function was used to perform UMAP dimensionality reduction on the cell clustering results, and the “plot1cell” package was used to visually display the distribution of different cell populations in PCa. For the expression of RAB26, we used the “Nebulosa” package to analyze and visualize the expression density of RAB26 in different cell types. Then, we downloaded pan-cancer RNA sequencing data and corresponding sample clinical information from The Cancer Genome Atlas (TCGA) database and used R packages such as “TCGAbiolinks” and “limma” for normalization processing and differential expression analysis, and “ggplot2” for visualization. To explore the correlation between RAB26 and the prognosis of PCa patients, we used the “survival” package for survival analysis and used the Kaplan–Meier method and log-rank test to evaluate survival rate differences.

### Statistical analysis

In all cases, data were presented as mean ± standard deviation. GraphPad Prism v. 8.02 (GraphPad Software Inc., San Diego, CA, USA) was used to conduct statistical analysis. Analysis of variance, *t*-test, or *χ*^2^-test was used to detect differences between groups. *p*-values <0.05 were considered statistically significant.

## Results

### Overexpression of RAB26 in PCa is associated with poor prognosis

**Figure 1:**
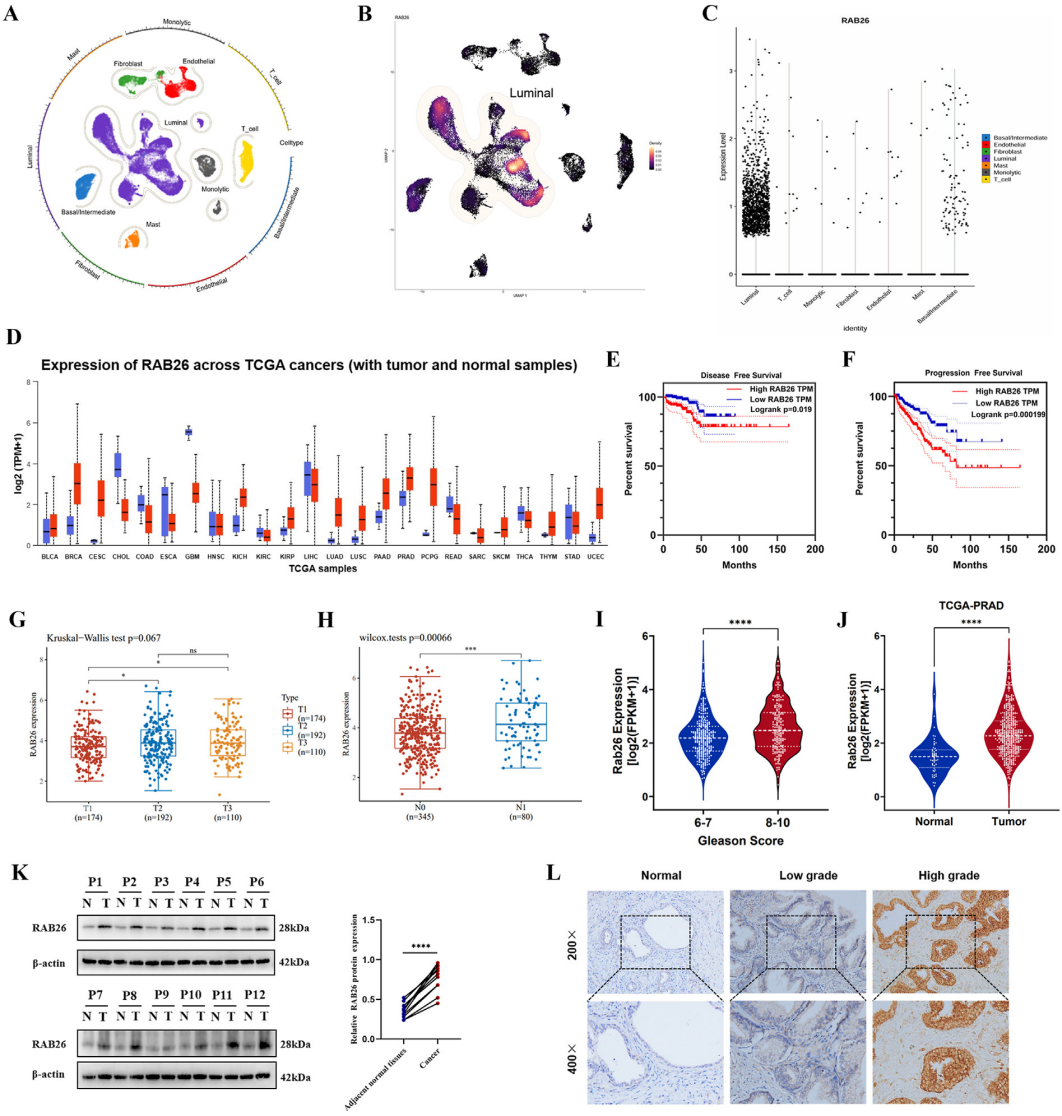
Analysis of clinical correlation between RAB26 expression in prostate cancer. **(A)** Expression density map of single-cell sequencing cell typing in prostate cancer. **(B)** UMAP diagram of RAB26 in prostate cancer cells. **(C)** Expression of RAB26 in different cell types. **(D)** Expression of RAB26 in different tumors. **(E)** RAB26 and progression-free survival. **(F)** RAB26 and disease-free survival. **(G)** The relationship between RAB26 and the T stage of prostate cancer. **(H)** The relationship between RAB26 and lymph node metastasis in prostate cancer. **(I)** The relationship between RAB26 and different Gleason scores. **(J)** Differential expression of RAB26 between normal prostate tissue and prostate cancer tissue. **(K)** Western blotting detected RAB26 in the tumor and para-carcinoma tissues of patients with prostate cancer. **(L)** The immunohistochemical staining of RAB26 in patients with prostate cancer. ∗*p* < 0.05, ∗∗*p* < 0.01, ∗∗∗*p* < 0.001, and ∗∗∗∗*p* < 0.0001.

To determine whether RAB26 had any clinical significance, we first downloaded the PCa single-cell sequencing data (GSE141445) and analyzed the expression and distribution of RAB26 in the data. The results indicated that seven primary cell groups, namely, luminal cell, basal/intermediate cell, T cell, endothelial cell, fibroblast, monocyte, and mast cell, were identified (Fig. 1A). Subsequent efforts were made to ascertain the cellular source of RAB26 expression. Violin and UMAP plots were utilized to demonstrate the differential expression of RAB26 across various cell clusters. Notably, RAB26 was mainly expressed by luminal and basal/intermediate cell clusters (Fig. 1B, C). To further investigate the expression of RAB26 in tumors, a pan-cancer analysis of RAB26 was conducted through the TCGA database. We discovered that RAB26 was up-regulated in multiple cancers (Fig. 1D). Then, we performed a Kaplan–Meier analysis to evaluate the prognostic efficacy of RAB26 in PCa. Concerning disease-free survival and progression-free survival, patients in the RAB26 high-expression group had a notably unfavorable prognosis (*p* < 0.001) (Fig. 1E, F). The results of the Kruskal–Wallis test and Wilcox test showed that RAB26 was significantly highly expressed in more advanced PCa (PCa with high T staging, N staging, and Gleason scores) (Fig. 1G–I). The findings from the TCGA-PRAD cohort of PCa patients showed that RAB26 was significantly highly expressed in PCa (Fig. 1J). Furthermore, we collected the cancerous and adjacent tissues and clinical information of 12 patients with PCa (Supplementary Table 1). The results obtained from the western blotting and immunohistochemistry experiments on clinically collected samples confirmed the up-regulation of RAB26 in the PCa group, with a more pronounced increase observed in high-grade PCa tissues (Fig. 1K). Based on the immunohistochemical staining intensity, we divided PCa patients into a low group and a strong group to observe the correlation between RAB26 expression and pathological characteristics (Fig. 1L). Patients in the strong group exhibited higher age (*p* < 0.05), Gleason scores (*p* < 0.01), ISUP stage (*p* < 0.001), T stage (*p* < 0.01), metastasis (*p* < 0.05), and serum PSA content (*p* < 0.05) (Table 2).

**Table 2:** Correlation between RAB26 expression and pathological characteristics of prostate cancer.

Pathological characteristics	Immunohistochemical staining intensity		χ^2^	*p*-value
	Low (*n*, %)	Strong (*n*, %)		
Age (years)				
<60	5 (2.9)	7 (4.1)	4.881	0.027
≥60	26 (15.1)	134 (77.9)		
Gleason score				
≤7	27 (15.7)	85 (49.4)	6.906	0.008
>8	4 (2.3)	56 (32.6)		
ISUP grade				
Ⅰ grade	13 (7.6)	14 (8.1)	22.25	0.0002
Ⅱ grade	10 (5.8)	46 (26.7)		
Ⅲ grade	4 (2.3)	26 (15.1)		
Ⅳ grade	2 (1.2)	19 (11.1)		
Ⅴ grade	2 (1.2)	36 (20.9)		
T stage				
T1-2	28 (16.3)	91 (52.9)	6.761	0.009
T3-4	3 (1.7)	50 (29.1)		
Metastasis				
Yes	1 (0.5)	28 (16.3)	3.899	0.048
No	30 (17.4)	113 (65.7)		
PSA (ng/mL)				
≤4	5 (2.9)	6 (3.5)	5.985	0.014
>4	26 (15.1)	135 (78.5)		

### RAB26 promotes cell proliferation, invasion, and migration and suppresses cell apoptosis in PCa *in vitro*

**Figure 2:**
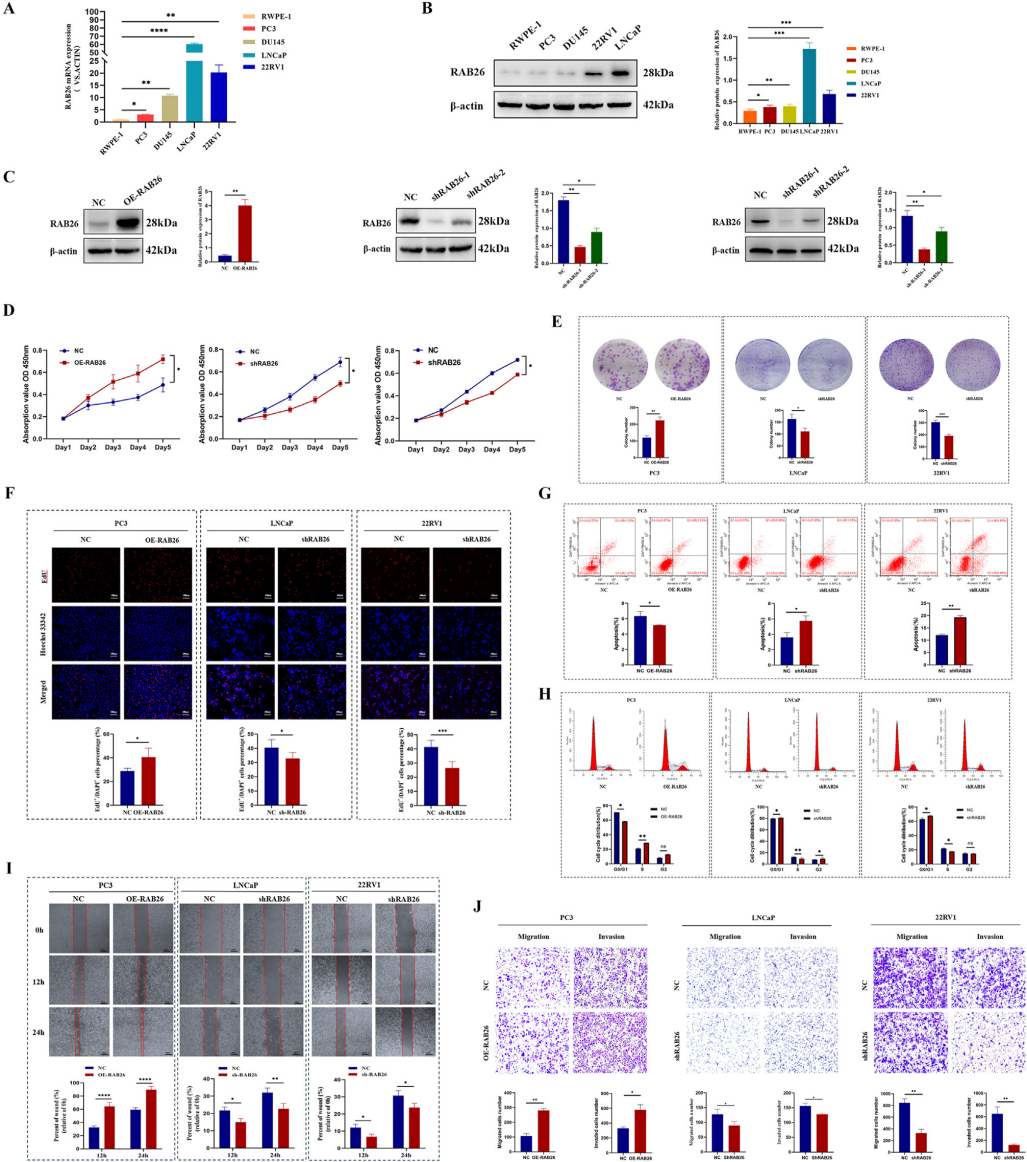
The function of RAB26 was confirmed by *in vitro* experiments. **(A, B)** Quantitative reverse-transcription PCR and western blotting detected the expression of RAB26 in different cell lines. **(C)** RAB26 was successfully overexpressed in PC3 cells and knocked down in LNCaP and 22RV1 cells. **(D)** The effect of RAB26 on the proliferation of PC3, LNCaP, and 22RV1 cells was evaluated using CCK-8 assay. **(E)** The effect of RAB26 on the ability of colony formation in PC3, LNCaP, and 22RV1 cells. **(F)** The effect of RAB26 on the proliferation of PC3, LNCaP, and 22RV1 cells was evaluated using EdU. **(G, H)** The effect of RAB26 on apoptosis and the cell cycle was detected by flow cytometry. **(I)** The effect of RAB26 on the migration and invasion of PC3, LNCaP, and 22RV1 cells was detected by transwell assay. **(J)** The effect of RAB26 on the migration of Caki-1 cells was detected by scratch test. ∗*p* < 0.05, ∗∗*p* < 0.01, ∗∗∗*p* < 0.001, and ∗∗∗∗*p* < 0.0001.

To evaluate the role of RAB26 in PCa progression, we detected the expression of RAB26 by quantitative reverse-transcription PCR and western blotting. It was found that RAB26 expression was higher in PCa cell lines than in RWPE-1, a normal prostate epithelial cell line (Fig. 2A, B), and RAB26 levels were highest in LNCaP cells and 22RV1 cells, and lowest in PC3 cells. Therefore, LNCaP cells and 22RV1 cells were used to establish stable RAB26 knockdown cells using lentiviral shRNAs, and PC3 cells were used to establish stable RAB26 overexpression cells using the lentiviral vector system. By western blotting, it was confirmed that RAB26 had been transfected successfully (Fig. 2C). The cell viability was examined by the CCK-8 assay. The results showed that cell viability decreased in LNCaP shRAB26 cells and 22RV1 shRAB26 cells but increased in PC3 RAB26 cells compared with control groups (Fig. 2D). Consistently, the colony formation assay indicated that the knockdown of RAB26 remarkably inhibited the growth capability of LNCaP and 22RV1 cells, and the overexpression of RAB26 promoted the growth capability of PC3 cells (Fig. 2E). To further clarify the effect of RAB26 on PCa cell proliferation, we performed EdU experiment. The results showed that the down-regulation of RAB26 expression could decrease the proliferation ability of LNCaP and 22RV1 cells and its up-regulation could increase the proliferation ability of PC3 cells (Fig. 2F). In addition, a flow cytometry analysis was performed to evaluate whether RAB26 affected PCa cells by altering the cell cycle profile and apoptosis. Apoptosis assays also revealed that the knockdown of RAB26 had an apoptosis-inducing effect on LNCaP and 22RV1 cells, and the overexpression of RAB26 inhibited apoptosis in PC3 cells (Fig. 2G). As well, compared with control groups, RAB26 significantly decreased LNCaP and 22RV1 cells in the S phase after RAB26 transfections and increased PC3 cells in the S phase (Fig. 2H).

Afterward, to evaluate the functions of RAB26 on cell migration and invasion *in vitro*, transwell and wound healing assays were conducted. Using the wound healing assay, we found that RAB26 knockdown inhibited the migration of LNCaP and 22RV1 cells, and RAB26 overexpression promoted the migration of PC3 cells (Fig. 2I). According to transwell migration and invasion assays, RAB26 knockdown significantly inhibited cell migration and invasion levels, and RAB26 overexpression promoted cell migration and invasion, as compared with control groups (Fig. 2J).

### RAB26 enriches the transcription of genes that promote EMT and regulates the stem cell-like phenotype in PCa cells

**Figure 3:**
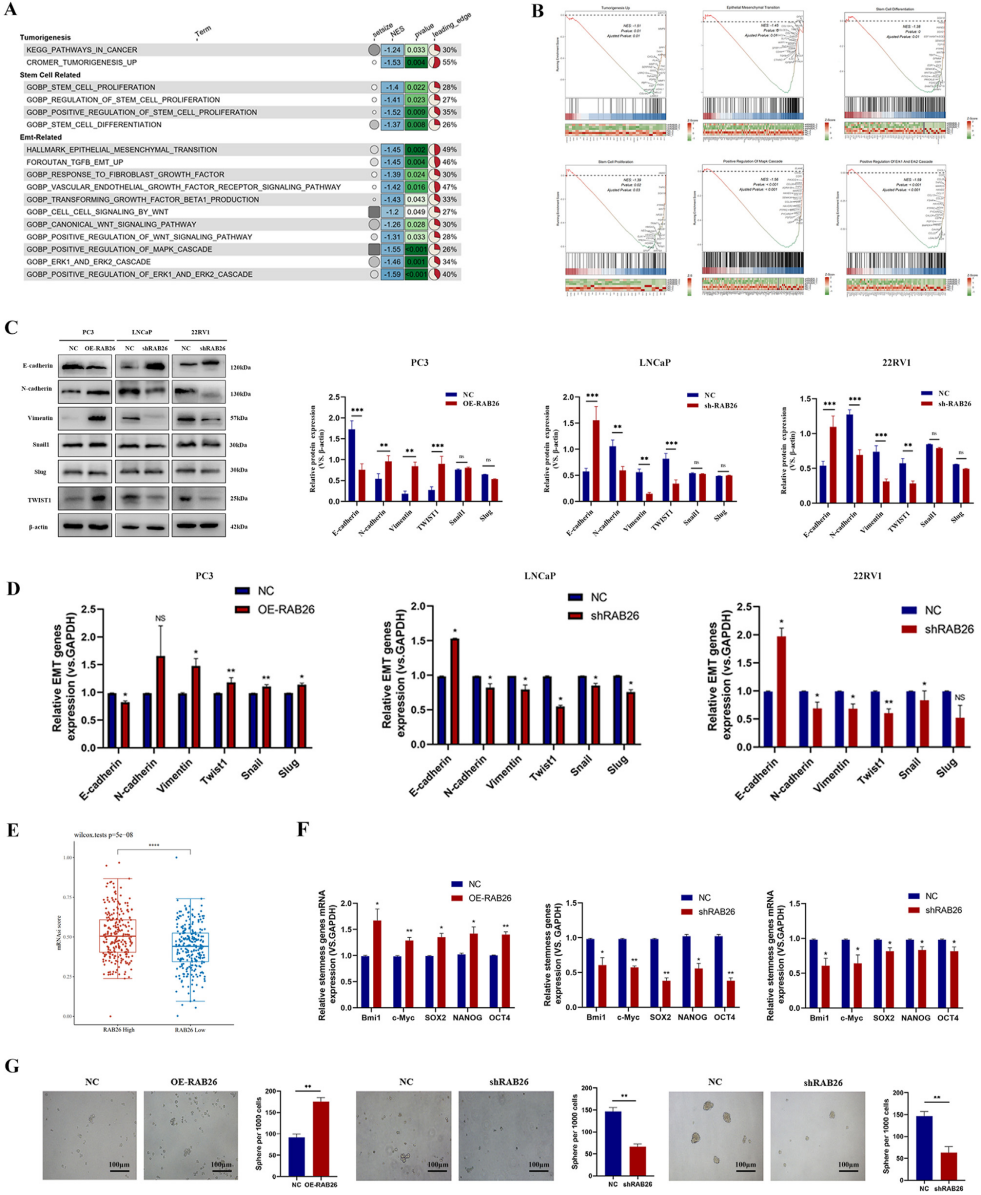
RAB26 promotes epithelial–mesenchymal transition and stem cell-like characteristics of prostate cancer cells. **(A, B)** The results of GSEA enrichment analysis and representative GSEA data. **(C, D)** Western blotting and quantitative reverse-transcription PCR detected the expression of epithelial-mesenchymal transition-related marker proteins in PC3, LNCaP, and 22RV1 cells. **(E)** The relationship between RAB26 and mRNA score. **(F)** Quantitative reverse-transcription PCR detected the expression of stem cell-related markers in PC3, LNCaP, and 22RV1 cells. **(G)** RAB26 affected the formation of tumor stem cell spheres in PC3, LNCaP, and 22RV1 cells. ∗*p* < 0.05, ∗∗*p* < 0.01, ∗∗∗*p* < 0.001, and ∗∗∗∗*p* < 0.0001.

To unveil the molecular mechanism underlying RAB26 affecting tumor proliferation and metastasis, we performed transcriptome sequencing analysis to compare transcriptome profiles between the knockdown group and the negative control group in LNCaP cells. Based on the differentially expressed gene analysis dataset, we performed gene set enrichment analysis (GSEA) (Fig. 3A). GSEA plots indicate significant enrichment of tumorigenesis up, stem cell differentiation, EMT, and stem cell proliferation in the knockdown RAB26 compared with control (Fig. 3B). Thus, to investigated whether RAB26 was associated with EMT, some EMT-related markers were detected by PCR and western blotting assays. The results demonstrated that E-cadherin was up-regulated while N-cadherin, vimentin, and TWIST1 were down-regulated following the knockdown of RAB26 in LNCaP and 22RV1 cells. The opposite results were obtained after RAB26 overexpression. However, protein expression of snail and slug had no significant effect in PCa cells (Fig. 3C, D). The combined findings indicate that RAB26 plays a vital role in tumor development and facilitates the process of EMT.

As a dedifferentiation potential marker, mRNA stemness indices (mRNAsi) are applied to tumor cells to measure their stemness. Therefore, we first investigated the correlation between the expression of RAB26 and the stemness score. The results showed that higher expression of RAB26 was associated with higher mRNAsi (Fig. 3E), suggesting RAB26 plays an important role in PCa progression. Then, the gene expression of the pluripotency marks Bmi1, c-Myc, SRY-box transcription factor 2 (SOX2), NANOG, and octamer-binding transcription factor 4 (OCT4) was demonstrated using PCR. The results found that overexpressing RAB26 cells highly expressed these genes, whereas knocking down RAB26 cells showed lower expression levels (Fig. 3F). In addition, to further investigate the potential biological function of RAB26 in the self-renewal of PCSCs, tumor sphere formation assays were performed. The results showed that up-regulating RAB26 increased tumor spheroid size and number in PC3 cells, whereas down-regulating RAB26 decreased them in LNCaP and 22RV1 cells (Fig. 3G). Therefore, our findings demonstrate that RAB26 could facilitate the development of stem-like characteristics in PCa.

### RAB26 promotes the nuclear translocation of ERK1/2 and up-regulation of TWIST1 transcription

**Figure 4:**
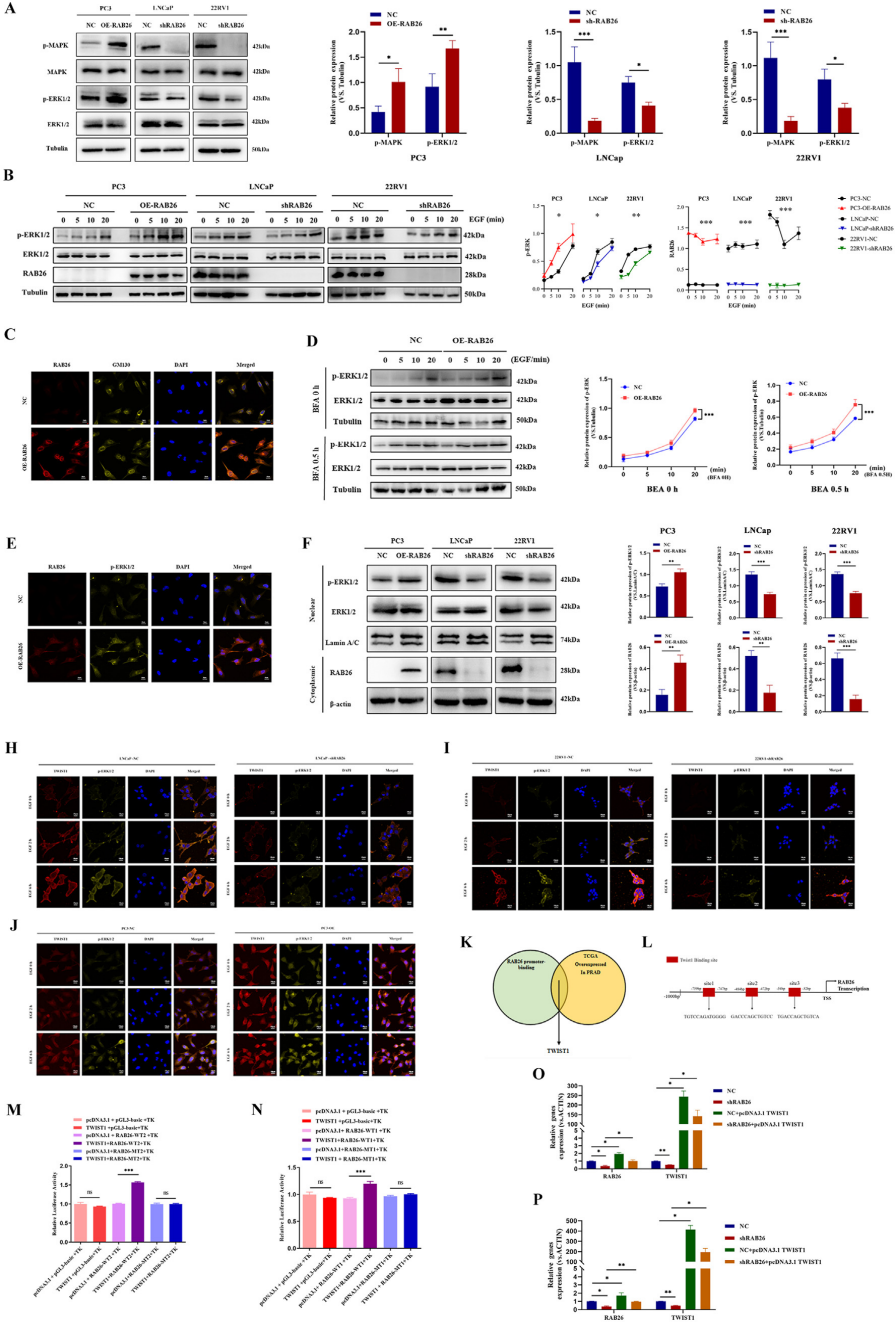
RAB26 promotes epithelial–mesenchymal transition and stem cell-like characteristics of prostate cancer cells via MAPK-ERK/TWIST1 axis. **(A)** Western blotting detected MAPK/ERK1/2 signaling pathway-related proteins in PC3, LNCaP, and 22RV1 cells. **(B)** After serum starvation, western blotting detected epidermal growth factor (EGF)-treated cells to determine the phosphorylation of ERK1/2 at a specified time point after serum starvation. **(C)** Immunofluorescence assay detected the binding condition of RAB26 (red) and GM130 (yellow). **(D)** Western blotting detected ERK1/2 and p-ERK1/2 in PC3 after brefeldin A (BFA) treatment. **(E)** Immunofluorescence assay detected the binding condition of RAB26 (red) and p-ERK1/2 (yellow). **(F)** Western blotting detected nucleocytoplasmic distribution of p-ERK1/2 and RAB26. **(G)** Western blotting detected nucleocytoplasmic distribution of p-ERK1/2, TWIST1, and RAB26. **(H**–**J)** Immunofluorescence assay detected RAB26/p-ERK1/2 promoting TWIST1 nuclear translocation in PC3, LNCaP, and 22RV1 cells. **(K)** Combined JASPAR and TCGA predicted transcription factors bound by RAB26 promoter. **(M, N)** A double luciferase gene reporting experiment detected luciferase activity. **(O, P)** Quantitative reverse-transcription PCR detected gene expression.

To explore the aberrant signaling that RAB26 may be involved in PCa, we performed GSEA to evaluate cancer-relevant signaling pathways, which are regulated by RAB26 expression alterations in PCa. We found that RAB 26 may regulate the aberrant conduction of the MAPK and ERK pathways in PCa (Fig. 3A, B). Therefore, we performed western blotting assays to confirm the protein expression levels of p-MAPK, MAPK, p-ERK1/2, and ERK1/2. The results showed that overexpression of RAB26 significantly increased MAPK and ERK1/2 phosphorylation levels, and RAB26 knockdown decreased the phosphorylation of MAPK and ERK1/2 without affecting the total levels of MAPK and ERK1/2 in PCa cells (Fig. 4A). To further investigate whether RAB26 could promote PCa progression by activating phosphorylated ERK1/2. After serum starvation and EGF (5 ng/mL) stimulation, RAB26 overexpression markedly increased ERK1/2 activation monitored by p-ERK1/2 in a time-dependent manner in PC3 cells, which was also shown in western blotting results (Fig. 4B). At the same time, as the results we observed by confocal immunofluorescence microscopy also revealed the co-localization of GM130 and RAB26 (Fig. 4C), we used brefeldin A (BFA; 5 ug/mL) to block the trafficking from the GM130 to the endoplasmic reticulum. Finally, we found that BFA treatment damaged the GM130 structure but did not affect ERK phosphorylation, which suggests that ERK1/2 activation is more likely to be independent of RAB26's trafficking function (Fig. 4D).

We further hypothesized that RAB26 promoted ERK1/2 signal transduction into p-ERK1/2, and played a role after entering the nucleus. Thus, we analyzed the protein localization of p-ERK1/2 and RAB26 using confocal immunofluorescence microscopy and found a strong co-localization of p-ERK1/2 and RAB26 (Fig. 4E). The results demonstrated that RAB26 played a role by promoting p-ERK1/2 to enter the nucleus to work. Then, we determined through nuclear cytoplasmic separation experiments whether RAB26 could promote the entry of p-ERK1/2 into the nucleus. The results found that RAB26 could activate the phosphorylation of ERK1/2, promoting its effect after nuclear entry (Fig. 4F).

MAPK signaling can promote the nuclear localization of the transcription factor TWIST1 and further activate EMT.^30^ Then, after serum starvation and EGF (5 ng/mL) stimulation, RAB26 overexpression markedly increased ERK1/2 activation monitored by p-ERK1/2 in a time-dependent manner in PC3 cells, which was also shown in western blotting results (Fig. 4G). Intriguingly, we found that the level of TWIST1 increased with the prolongation of EGF stimulation time in the nucleus. Subsequently, this phenomenon was further confirmed by confocal microscopy analysis (Fig. 4H–J). According to the above results, we found that the level of TWIST1 protein expression was the most obvious. Further screening the bound transcription factors for the RAB26 promoter by JASPAR and TCGA databases, we found that TWIST1 could bind to the promoter of RAB26 for transcriptional regulation (Fig. 4K, L). We then selected site 2 and site 3 for dual-luciferase assays, and the results showed that RAB26 promoter significantly binds to TWIST1 (Fig. 4M, N). Furthermore, after overexpressing TWIST1 with plasmids, we found an increase in the transcription of RAB26 (Fig. 4O, P). The above results indicate that there may be a positive feedback expression regulation mechanism between RAB26 and TWIST1.

### RAB26 promotes the growth and metastasis of PCa cells *in vivo*

**Figure 5:**
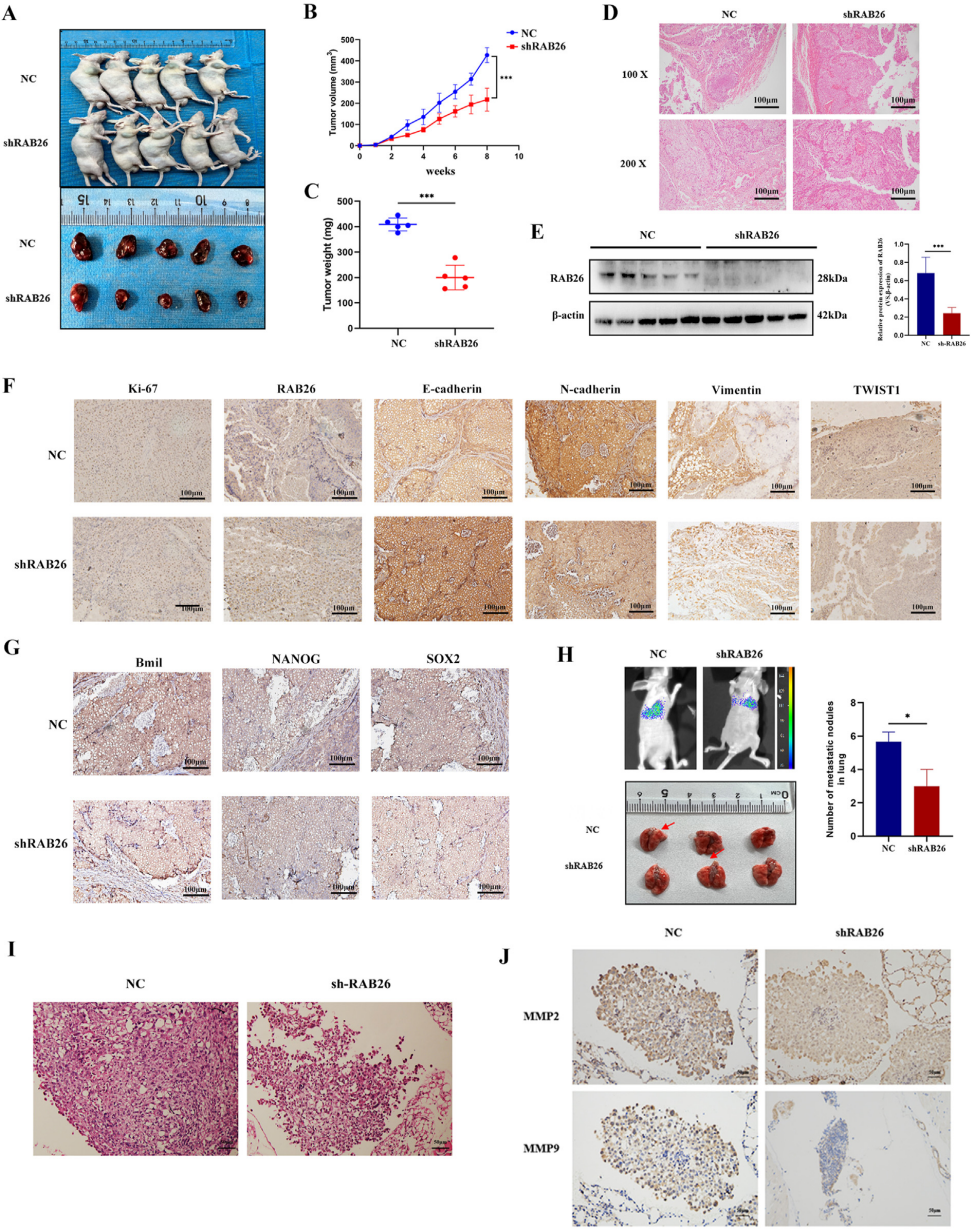
RAB26 promotes prostate cancer proliferation and metastasis *in vivo*. **(A)** Gross view of mice and tumors. **(B)** Growth curve of tumor volume. **(C)** The weight of the tumor. **(D)** Representative hematoxylin-eosin staining images in the xenograft model of prostate cancer. **(E)** Western blotting verified the expression of RAB26 in tumor tissue. **(F)** Representative immunohistochemistry images of epithelial-mesenchymal transition-related markers in tumor tissue. **(G)** Representative immunohistochemistry images of Bmil, NANOG, and SOX2 in tumor tissue. **(H)** Imaging of living small animals and gross images of lung metastases. **(I)** Representative hematoxylin-eosin staining images of a lung metastatic tumor. **(J)** Representative immunohistochemistry images of MMP2 and MMP9 in lung metastatic tumor. ∗*p* < 0.05, ∗∗*p* < 0.01, ∗∗∗*p* < 0.001, and ∗∗∗∗*p* < 0.0001.

To further confirm the effects of RAB26 *in vivo*, we established a BALB/c nude mouse xenograft model using LNCaP cells. After 8 weeks, we found that tumors with RAB26 knockdown displayed significantly reduced growth rates compared with control tumors (Fig. 5A, B), and the tumor weight of the mice in the RAB26 knockdown group was significantly lower than that of the control group (Fig. 5C). Next, tumors were cut into sections and stained with hematoxylin and eosin. The results were consistent with the characteristics of adenocarcinoma (Fig. 5D). Western blotting analysis revealed a significant reduction in RAB26 expression in tumors from the knockdown group (Fig. 5E). Subsequently, we examined Ki-67, RAB26, E-cadherin, N-cadherin, vimentin, TWIST1, Bmi1, NANOG, and SOX2 protein expression by immunohistochemical staining. The results demonstrated that, compared with the control group, RAB26 knockdown decreased the positive rate of Ki-67, RAB26, N-cadherin, vimentin, TWIST1, Bmi1, NANOG, and SOX2 and increased the positive rate of E-cadherin (Fig. 5F, G), suggesting that RAB26 expression was related to tumor growth, EMT, and stemness.

To further determine the role of RAB26 in PCa metastasis *in vivo*, we developed a nude mouse model using LNCaP cells through tail vein injections. After 12 weeks, small animal live imaging showed that knocking down RAB26 could significantly reduce the level of tumor metastasis. We found that the number of lung metastases in the knockdown RAB26 group decreased compared with the control group (Fig. 5H). Hematoxylin-eosin staining results indicated normal structural destruction of the lungs, deep staining of nuclei, and enlargement of the nucleoli (Fig. 5I). RAB26 knockdown decreased the expression of matrix metalloproteinase 2/9 (MMP2/9), compared with shRAB26-NC (Fig. 5J). Taken together, these results suggest that RAB26 promotes PCa metastasis *in vivo*.

## Discussion

PCa is one of the most common malignant tumors in men worldwide, which seriously endangers men's health. The most common treatments for PCa now are androgen deprivation therapy and surgery, but the occurrence of drug resistance and distant metastasis still brings difficulties to the cure of PCa. Cancer stem cells are the root cause of tumorigenesis, as well as a significant mechanism for tumor metastasis, drug resistance, and recurrence after treatment.^31^ The cancer stem cell hypothesis holds that cancer stem cells can rapidly divide into amplified and differentiated tumor cells, thereby promoting tumor growth.^32^

Our research first analyzed the differential genes between normal prostate cells, PCa cells, and PCSCs through bioinformatics. We found that RAB26 was highly expressed in both PCa cells and PCSCs. Through single-cell sequencing analysis, we found that RAB26 was mainly expressed by luminal and basal/intermediate cell clusters. Research has shown that luminal cells are a group of castration-resistant prostate cells, and it has been confirmed that luminal cells are castration-resistant prostate stem cells, which may serve as prostate tumor progenitor cells.^32^ This indicates that the origin of RAB26 may originate from luminal cells and be involved in castration-resistant PCa progression. Therefore, we speculate that RAB26 may be a key molecule leading to PCa recurrence, metastasis, and drug resistance.

Furthermore, through analysis of the TCGA database, it is indicated that RAB26 is a key molecule affecting poor prognosis in patients with PCa. Additionally, we demonstrated that RAB26 played a role in promoting tumor proliferation, invasion, and migration in PCa. Meanwhile, RAB26 promoted the spheroidization ability of PCa cells. This result is consistent with the role of RAB26 in non-small cell lung cancer.^33^ However, in breast cancer, RAB26 inhibits the migration and invasion of breast cells by mediating autophagic degradation and phosphorylation of Src.^28^ These differential results regarding the impact of RAB26 on tumor development may be due to potential tumor-specific differences.

The RAB family plays a key role in epithelial neoplasia, and its high expression in cancer is often associated with cancer metastasis and progression.^34^, ^35^, ^36^ Studies have shown that RAB2A can directly interact with MAPK phosphatase and prevent the dephosphorylation of Erk1/2, which enhances the tumorigenicity of breast cancer stem cells. Activation of the MAPK/ERK pathway normally promotes tumor progression and is a hallmark of many cancers. Thus, the components and regulators of the ERK pathway have been proposed as potential therapeutic targets in cancer.^37^^,^^38^ Increasing evidence has shown that activation of the ERK signaling pathway will promote PCa metastasis. Bloom syndrome protein (BLM) regulates the activation of the MAPK/ERK signaling pathway by interacting with hepatoma-derived growth factor (HDGF), inducing KRAS expression and RhoA inhibition to promote the malignant progression of PCa.^39^ Hsa_circ_0003258 can bind to insulin-like growth factor 2 mRNA binding protein 3 (IGF2BP3), which enhances the stability of histone deacetylase 4 (HDAC4), thereby activating the ERK signaling pathway, promoting EMT, and ultimately accelerating PCa metastasis.^40^ EMT is involved in the occurrence and development of tumors, including tumor initiation, malignant progression, tumor dryness, tumor cell migration, blood infiltration, metastasis, and treatment resistance. It is considered an important mechanism regulating the initial steps of cancer metastasis and progression.^41^^,^^42^ EMT is typically characterized by the loss of the epithelial markers E-cadherin and β-catenin, accompanied by increased cell migration and invasiveness due to the activation of specific transcription programs.^43^ EMT plays an important role in the development and metastasis of PCa and may be a key target for PCa treatment.^44^ Our results suggested that RAB26 activated MAPK/ERK phosphorylation and increased the expression of EMT-related markers E-cadherin, N-cadherin, vimentin, and TWIST1. In addition, p-ERK1/2 levels increased significantly in a time-dependent manner after serum starvation and EGF stimulation. p-ERK1/2 was similarly increased in RAB26-overexpressing PCa cells, with opposite results after RAB26 knockdown, suggesting that RAB26 promotes EMT through the MAPK/ERK signaling pathway in PCa.

It is well known that RAB26 has a vesicle transport function.^45^ To clarify whether the vesicle transport function of RAB26 was related to ERK activation, we used 10 μg/mL BFA to block protein transport from the endoplasmic reticulum to the Golgi apparatus. Our study found that BFA blocked vesicle trafficking, but the phosphorylation of ERK1/2 was not affected, suggesting that the activation of ERK1/2 may not be dependent on the transport function of RAB26. To further explore the molecular mechanism underlying RAB26 promoting EMT in PCa through ERK signaling, we used confocal immunofluorescence staining to confirm that RAB26 co-localized with p-ERK1/2 and RAB26 could promote p-ERK entry into the cell nucleus. The initiation of ERK 1/2 entry into the nucleus is often induced by phosphorylation at position Thr202, Tyr204/Thr185, and Tyr187.^46^^,^^47^ Whether this biological process participates in RAB26-mediated abnormal transcriptional regulation requires more research to clarify. TWIST1 is a transcription factor with strong EMT-inducing ability.^17^ In liver cancer, COMP/CD36 signaling causes phosphorylation of AKT and ERK, which leads to the up-regulation of EMT-related markers such as TWIST1, Slug, and MMP9.^48^ α-Linolenic acid inhibits the migration of human triple-negative breast cancer by attenuating the phosphorylation of c-Jun N-terminal kinase (JNK), ERK, and Akt, reducing TWIST1 expression, and inhibiting TWIST1-mediated EMT.^49^ It has been shown that TWIST can accelerate cellular EMT, leading to the progression of PCa.^50^ Our results found that the levels of TWIST1 in the nucleus increased with the prolongation of EGF stimulation time, indicating that ERK activation could promote TWIST1 entry into the nucleus. Meanwhile, in the presence of RAB 26 overexpression, TWIST1 protein levels in the nucleus increased notably, but the result was reversed after knockdown. Interestingly, after the expression of TWIST1 increases, the transcription of RAB26 also increases accordingly, ultimately forming a positive feedback regulation to promote PCa progression. Overall, our study demonstrates that RAB26 activates the nuclear localization of TWIST1 through the MAPK/ERK pathway, and further, TWIST1 binds to the promoter of RAB26 to promote the transcription of RAB26.

Although our results found that RAB26 could affect the stemness of PCSCs, activate the ERK signaling pathway, up-regulate the expression of TWIST1, and form a positive feedback regulation between the ERK signaling pathway and TWIST1 to enhance EMT and promote the malignant progression of PCa, we did not further explore the specific phosphorylation site of ERK.

## Conclusion

RAB26 is overexpressed in PCa tissues and is closely associated with poor prognosis, Gleason score, ISUP grading, metastasis, PSA, and clinical T stage. RAB26 promotes the proliferation, migration, and invasion of PCa cells, inhibits apoptosis, and enhances the stemness and sphere formation of PCSCs. Further studies revealed that RAB26 could facilitate p-ERK-mediated TWIST1 nuclear translocation, thereby regulating the epithelial–mesenchymal transition and stemness in PCa cells. *In vivo*, experiments confirmed the significant promotion of PCa growth and metastasis by RAB26 overexpression. Overall, this study provides a comprehensive understanding of the functional and molecular mechanisms of RAB26 in PCa, offering new perspectives and potential targets for molecularly targeted therapy of PCa.

## CRediT authorship contribution statement

**Hexi Wang:** Writing – original draft, Data curation. **Simin Liang:** Methodology. **Xiaoyi Du:** Software. **Guozhi Zhao:** Data curation. **Yuanyuan Bai:** Methodology. **Junwu Li:** Methodology. **Haoyu Xu:** Visualization. **Senlin Peng:** Writing – review & editing. **Wei Tang:** Project administration.

## Ethics declaration

All samples and clinical data were collected from the First Affiliated Hospital of Chongqing Medical University. Patient consents were obtained to use these clinical specimens for research purposes. Our study was approved by the Ethics Committee of the First Affiliated Hospital of Chongqing Medical University according to the 1975 Declaration of Helsinki. All animal experiments were approved by the Institutional Animal Care and Use Committee of Chongqing Medical University. All contributing authors agree to the publication of this article.

## Data availability

All data are fully available without restriction.

## Funding

This work was supported by grants from the 10.13039/501100002865Chongqing Science and Technology Bureau (China) (No. CSTB2022BSXM-JCX0040).

## Conflict of interests

The authors declared no competing interests.
