# Supplementary material for: RAB26 promotes prostate cancer progression via the MAPK/ERK-TWIST1 signaling axis
Source: Genes Dis. 2025 May 22;12(6):101689. doi: 10.1016/j.gendis.2025.101689 (PMC12357246; doi:10.1016/j.gendis.2025.101689)
Supplement: Multimedia component 1 [file mmc1.docx]

**Supplementary Table 1. Baseline data table of the 12 patients whose surgical specimens were collected**

| **Clinicopathological characteristics** | **N（%）** |
| --- | --- |
| **Age（years）** |  |
| <60 | 4（33.3） |
| ≥60 | 8（66.7） |
| **Gleason score** |  |
| ≤7 | 5（41.7） |
| >8 | 7（58.3） |
| **ISUP grade** |  |
| Ⅰ grade | 1（8.3） |
| Ⅱ grade | 4（33.3） |
| Ⅲ grade | 5（41.7） |
| Ⅳ grade | 2（16.7） |
| Ⅴ grade | 0（0） |
| **T stage** |  |
| T1-2 | 7（58.3） |
| T3-4 | 5（41.7） |
| **Metastasis** |  |
| Yes | 0（0） |
| No | 12（100） |
| **PSA (n*g*/ml)** |  |
| ≤4 | 2（16.7） |
| >4 | 11（83.3） |
